# Assessing Discharge Communication and Follow-up of Acute Kidney Injury in British Columbia: A Retrospective Chart Review

**DOI:** 10.1177/20543581231222064

**Published:** 2024-02-05

**Authors:** Peter Birks, Bader Al-Zeer, Daniel Holmes, Rami Elzayat, Mark Canney, Ognjenka Djurdjev, Tianyi Selena Shao, Yuyan Zheng, Samuel A. Silver, Adeera Levin

**Affiliations:** 1 Division of Nephrology, The University of British Columbia, Vancouver, Canada; 2 Faculty of Medicine, The University of British Columbia, Vancouver, Canada; 3 Department of Pathology and Laboratory Medicine, The University of British Columbia, Vancouver, Canada; 4 Department of Medicine, University of Ottawa, Ontario, Canada; 5 BC Renal, Vancouver, Canada; 6 Department of Medicine, Queen’s University, Kingston, Ontario, Canada

**Keywords:** acute kidney injury, acute kidney failure, kidney disease progression, nephrology quality improvement, nephrology discharge communication

## Abstract

**Background and objective:**

Acute kidney injury (AKI) affects up to 20% of hospitalizations and is associated with chronic kidney disease, cardiovascular disease, increased mortality, and increased health care costs. Proper documentation of AKI in discharge summaries is critical for optimal monitoring and treatment of these patients once discharged. Currently, there is limited literature evaluating the quality of discharge communication after AKI. This study aimed to evaluate the accuracy and quality of documentation of episodes of AKI at a tertiary care center in British Columbia, Canada.

**Methods, design, setting, patients, and measurements:**

This was a retrospective chart review study of adult patients who experienced AKI during hospital admission between January 1, 2018, and December 31, 2018. Laboratory data were used to identify all admissions to the cardiac and general medicine ward complicated by AKI defined by the Kidney Disease Improving Global Outcomes (KDIGO) criteria. A random sample of 300 AKI admissions stratified by AKI severity (eg, stages 1, 2, and 3) were identified for chart review. Patients were excluded if they required ongoing renal replacement therapy after admission, had a history of kidney transplant, died during their admission, or did not have a discharge summary available. Discharge summaries were reviewed for documentation of the following: presence of AKI, severity of AKI, AKI status at discharge, practitioner and laboratory follow-up plans, and medication changes.

**Results:**

A total of 1076 patients with 1237 AKI admissions were identified. Of the 300 patients selected for discharge summary review, 38 met exclusion criteria. In addition, AKI was documented in 140 (53%) discharge summaries and was more likely to be documented in more severe AKI: stage 1, 38%; stage 2, 51%; and stage 3, 75%. Of those with their AKI documented, 94 (67%) documented AKI severity, and 116 (83%) mentioned the AKI status or trajectory at the time of discharge. A total of 239 (91%) of discharge summaries mentioned a follow-up plan with a practitioner, but only 23 (10%) had documented follow-up with nephrology. Patients with their AKI documented were more likely to have nephrology follow-up than those without AKI documented (17% vs 1%). Regarding laboratory investigations, 92 (35%) of the summaries had documented recommendations. In summaries that included medications typically held during AKI, only about half made specific reference to those medications being held, adjusted, or documented a post-discharge plan for that medication. For those with nonsteroidal anti-inflammatory drugs (NSAIDs) listing, 64% of discharge summaries mentioned holding, and 9% mentioned a discharge plan. For those with angiotensin converting enzyme inhibitor (ACEi)/angiotensin II receptor blocker (ARB) listing, 38% mentioned holding these medications, and 46% mentioned a discharge plan. In summaries with diuretics listed, 35% mentioned holding, and 51% included a discharge plan.

**Conclusions and limitations:**

We found suboptimal quality and completeness of discharge reporting in patients hospitalized with AKI. This may contribute to inadequate follow-up and post-hospitalization care for this patient population. Strategies are required for increasing the presence and quality of AKI reporting in discharge summaries. Limitations include our definition of AKI based on lab criteria, which may have missed some of the injuries that met the criteria based on urine output. Another limitation is that our definition of AKI based on the highest and lowest creatinine during admission may have led to some overclassification. In addition, without outpatient laboratories, it is possible that we have not captured the true baseline creatinine in some patients.

## Abrégé

**Contexte et objectif:**

L’insuffisance rénale aiguë (IRA) complique jusqu’à 20 % des hospitalisations; elle est associée à l’insuffisance rénale chronique, aux maladies cardiovasculaires, à une mortalité accrue et à une augmentation des coûts de santé. La documentation appropriée de l’IRA dans les résumés de départ est essentielle pour optimiser la surveillance et le traitement des patients après leur sortie de l’hôpital. Il existe peu de littérature évaluant la qualité de la documentation de l’IRA dans les résumés de départ. Cette étude visait à évaluer l’exactitude et la qualité de la documentation des épisodes d’IRA dans un center de soins tertiaires de la Colombie-Britannique (Canada).

**Méthodologie, conception et cadre de l’étude, sujets et mesures:**

Il s’agit d’une étude rétrospective des dossiers de patients adultes ayant présenté une IRA au cours de leur admission à l’hôpital entre le 1^er^ janvier 2018 et le 31 décembre 2018. Les données de laboratoire ont été utilisées pour répertorier toutes les admissions compliquées par une IRA (définie par les critères KDIGO) dans les services de cardiologie et de médecine générale. Un échantillon aléatoire de 300 admissions avec IRA stratifiée selon sa gravité (p. ex., stade, 1, 2 et 3) a été constitué pour l’examen des dossiers. Ont été exclus les patients qui avaient eu besoin d’une thérapie de suppléance rénale continue après leur admission, ceux qui avaient des antécédents de transplantation rénale, ceux qui étaient décédés pendant leur admission et ceux pour qui aucun résumé de départ n’était disponible. Les résumés de départ ont été examinés à la recherche d’une mention des éléments suivants : présence d’une IRA, gravité de l’IRA, statut de l’IRA à la sortie, plans de suivi pour les tests de laboratoire et suivi avec un praticien, changements dans la médication.

**Résultats:**

En tout, 1 076 patients avec un total de 1 237 admissions avec IRA ont été identifiés. Parmi les 300 patients sélectionnés pour l’examen du résumé de départ, 38 répondaient aux critères d’exclusion. L’IRA avait été documentée dans 140 (53 %) des cas et plus elle était grave, plus elle était susceptible d’être documentée (stade 1 = 38 %; stade 2 = 51 %; stade 3 = 75 %). Parmi ceux où l’IRA était documentée, 94 (67 %) mentionnaient sa gravité et 116 (83 %) mentionnaient son statut ou sa trajectoire à la sortie du patient. Un plan de suivi avec le praticien était mentionné dans 239 (91 %) des résumés de départ, mais seuls 23 (10 %) mentionnaient un suivi en néphrologie. Les patients dont l’IRA était documentée étaient plus susceptibles de faire l’objet d’un suivi en néphrologie que ceux sans mention de l’IRA (17 % contre 1 %). En ce qui concerne les plans de suivi de laboratoire, 92 (35 %) des résumés contenaient des recommandations. Dans les résumés qui mentionnaient des médicaments normalement maintenus pendant un épisode d’IRA, seule la moitié environ faisait spécifiquement référence à ces médicaments comme ayant été cessés, ajustés ou documentés dans un plan post-sortie. Dans les résumés de départ qui listaient des AINS, 64 % mentionnaient qu’ils avaient été cessés temporairement et 9 % comprenaient un plan au congé de l’hôpital. Dans les résumés de départ qui listaient des IECA/ARA, 38 % mentionnaient que ces médicaments avaient été cessés temporairement et 46 % comprenaient un plan au congé de l’hôpital. Dans les résumés qui listaient des diurétiques, 35 % mentionnaient qu’ils avaient été cessés temporairement et 51 % comprenaient un plan au congé de l’hôpital.

**Limites et conclusion:**

Nous avons constaté que la qualité et l’exhaustivité des résumés de départ étaient sous-optimales chez les patients hospitalisés ayant vécu un épisode d’IRA. Cette situation peut contribuer à l’inadéquation du suivi et des soins post-hospitalization pour cette population de patients. Des stratégies sont nécessaires pour accroître la documentation d’un épisode d’IRA dans les résumés de départ et augmenter la qualité de sa communication. Les résultats de cette étude sont notamment limités par notre définition de l’IRA fondée sur des critères de laboratoire qui pourraient avoir manqué des patients répondant aux critères fondés sur la production d’urine. Notre définition de l’IRA fondée sur le taux de créatinine le plus élevée et le plus faible pendant l’admission pourrait également avoir conduit à un surdiagnostic. En outre, sans les résultats de laboratoires externes, il est possible que nous n’ayons pas saisi la mesure initiale réelle de la créatinine chez certains patients.

## Introduction

Acute kidney injury, defined as a rapid reduction in kidney function, is a commonly encountered complication among hospitalized patients.^1,2^ Patients who develop an AKI are at higher risk of developing chronic kidney disease (CKD), cardiovascular disease, hypertension, stroke, and mortality.^1,2^ These patients also experience lower quality of life and their care contributes to costs to the health care system.^3,4^ Recognizing episodes of AKI and ensuring patients who develop an AKI have adequate follow-up is important to detect early decline in kidney function and prevent further deleterious downstream effects.

There is growing evidence that targeted interventions in the follow-up period following hospital discharge is associated with decreased mortality and improved outcomes.^5,6^ These interventions include medication review and laboratory monitoring. As a result, Kidney Disease Improving Global Outcomes (KDIGO) have issued guidelines to improve patient follow-up, which include investigations at 3 months after AKI, to screen for resolution, and for evidence of new onset CKD or worsening of already existing CKD.^7^ However, despite these guidelines, there is generally poor follow-up and management of episodes of AKI following hospital discharge.^3,8-10^ The AKI documentation in discharge summaries is an important part of improving follow-up after AKI and has been shown to be associated with lower mortality.^5^

Unfortunately, documentation in discharge summaries has been found to be poor, including one study of patients with AKI showing that less than half of hospital discharge summaries mentioned the presence of AKI.^8^ Poor documentation limits the ability for patients to be adequately assessed for ongoing kidney dysfunction and may reduce referrals to a nephrologist who may be best suited to manage patients with more severe episodes of AKI.^3,11^ It has been shown that the quality of AKI documentation can improve with targeted interventions, such as adding a specific section to the discharge summary template to prompt documenting of AKI and using an AKI warning sticker for drug charts to remind the discharging doctor that the patient had an AKI during their stay.^9^

This study aimed to evaluate the accuracy and quality of documentation of episodes of AKI at St. Paul’s Hospital (SPH), a tertiary care center in British Columbia, Canada. The discharge summaries of these admissions were assessed for their quality of AKI documentation, including the stage, cause, details around medications and course. We hypothesized that deficits would be identified in the quality of discharge reporting of AKI, which can be used to guide future interventions to improve documentation, follow-up, and overall patient care.

## Methods

### Study Design, Setting, and Participants

This was a retrospective study of hospital discharge summaries. Ethical approval for this project was obtained through the University of British Columbia Research Ethics Board (REB: no. H19-01687). The study population comprised adult patients (age >18 years) who experienced non-dialysis-dependent AKI during admission to SPH cardiac and general medicine wards between January 1, 2018, and December 31, 2018. Of those patients identified, a random sample of 300 patients was chosen to perform an in-depth chart review of their discharge summaries. A total of 300 patient admissions were randomly selected by using simple random sampling without replacement using PROC SURVEYMEANS. Patients were excluded from the chart review if they were on renal replacement therapy prior to or during the admission, had a history of kidney transplant, died during their hospital admission, or did not have a discharge summary available.

### Data Sources and Definitions

We identified all hospital admissions to the general medicine and cardiac wards at SPH between January 1, 2018 and December 31, 2018. We focused on these wards because the probability of patients suffering AKI was thought to be highest. Laboratory data from the 2018 calendar year was extracted from an Oracle SQL mirror of the Sunquest Laboratory Information System. A patient admission was defined based on the following criteria: (1) new laboratory tests performed in the emergency room, postoperative recovery unit, or intensive care unit; (2) these new laboratory tests had to occur at least 1 week after any previous values (to assure that this represented a new hospital admission as opposed to follow-up tests from a prior admission); and (3) these initial laboratory tests needed to be followed by subsequent values within 48 hours from the cardiac or general medicine ward. This follows the logic that patients enter the hospital from the emergency, operating room, or critical care in the majority of cases and will have repeat tests once they reach the medical or cardiac unit. A patient discharge was defined as the absence of any laboratory tests from the ward for 2 weeks and the absence of laboratory tests from the emergency room in the same time period. All patients with laboratory tests drawn from hemodialysis or peritoneal dialysis unit locations during their admission were excluded.

Acute kidney injury was defined using the KDIGO creatinine criteria. During an admission, AKI was defined as an increase from baseline to highest creatinine in a 7-day window representing a 1.5-fold increase from baseline or an absolute rise of >26.5 µmol/L within 48 hours. Baseline creatinine was defined as the lowest creatinine value available during the admission. If the higher creatinine came before the lower creatinine, we still counted this as an AKI as it represents a kidney injury recovery. We only allowed one AKI to be counted per admission. Severity of AKI was categorized as per KDIGO staging with stage 1 (mild AKI) being creatinine 1.5 to 1.99 times baseline, stage 2 (moderate AKI) being 2.0 to 2.99 times baseline, and stage 3 (severe AKI) being ≥3.0 times baseline or an absolute rise in creatinine greater than 353.6 µmol/L or receipt of kidney replacement therapy.

### Discharge Summary Identification

A random sample of 300 patients having laboratory evidence of AKI was selected for chart review. The sample was stratified by AKI severity to ensure that each AKI severity stage was equally represented as we hypothesized that the severity may influence the likelihood of discharge reporting. The sample was also stratified by season (July-September, October-December, January-March, and April-June) because we hypothesized that the quality of discharge reporting in a tertiary setting may vary depending on the time of year, reflecting the timing of new trainees entering the hospital each July. During the discharge summary review process, if a patient was found to have received dialysis, died, or did not have a discharge summary, they were excluded.

### Discharge Summary Review for the Presence and Quality of AKI Communication

Specific criteria were outlined for reviewing the discharge summaries and data were collected on a standardized form (Supplemental Figure S1). The three reviewers included a nephrologist, an internal medicine resident, and a medical student. Twenty discharge summaries were initially reviewed by all 3 reviewers, and any discrepancies were resolved by consensus. The remaining summaries were split among the reviewers. Any uncertainties beyond the standardized data collection process were discussed among all reviewers and consensus was reached. Each discharge summary was reviewed in depth for the presence or absence of documentation of the following elements: the presence of AKI, severity of AKI, and AKI status at discharge. The discharge summaries were reviewed for documentation of follow-up plans, including whether any follow-up plan was mentioned and who the follow-up was with (primary care physician, other specialist, or nephrologist). We also assessed for documentation of a laboratory follow-up plan, including whether follow-up laboratories were recommended, what type of laboratories were recommended, the timeline for laboratory follow-up, and whether who was responsible to follow-up with the laboratories was specified. Finally, summaries were reviewed for recommendations around medication management.

### Statistical Analysis

The algorithms described previously for finding probable admissions and within-admission AKIs were programmed using R version 3.6.2. The analysis was conducted using SAS, version 9.4 (SAS Institute, Cary, North Carolina). Furthermore, χ^2^ test was used to compare laboratory recommendations among patients with and without AKI documentation in the discharge summary. Univariate logistic regression model between AKI stages and AKI documentation was used to calculate odds ratios (ORs) and *P* values. All graphic presentations, including bar graphs and pie charts, were created by MS Excel 2013.

## Results

### Participant Selection

**Figure 1.**
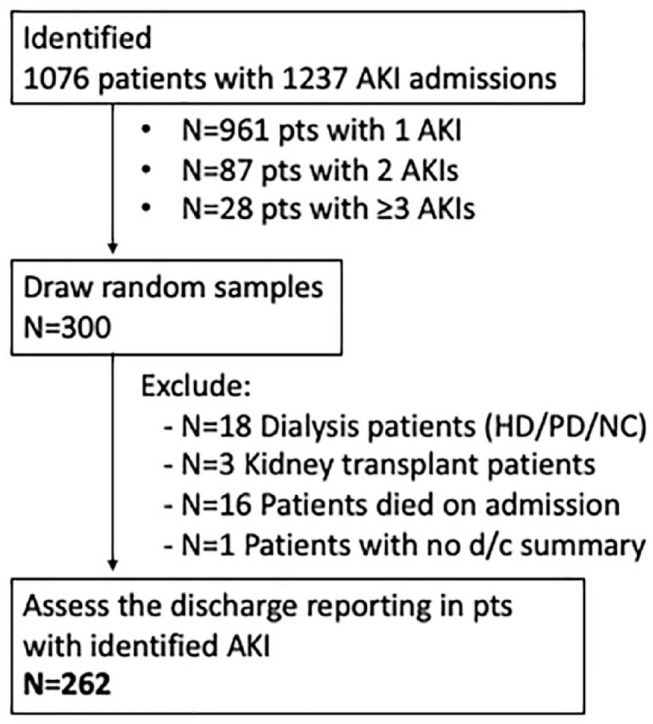
Discharge summary reports identification and screening. *Note*. AKI = acute kidney injury; HD = hemodialysis; PD = peritoneal dialysis; NC = nocturnal dialysis.

We identified 1076 patients with AKI, and 1237 distinct AKIs associated with admissions as some patients were admitted more than once. Of these AKI admissions, 800 (65%) were AKI stage 1; 264 (21%) stage 2; and 173 (14%) were AKI stage 3 (Table 1). Three hundred patients were randomly selected for in-depth review of discharge summary. Thirty-eight of these patients were excluded after review of their discharge summary revealed that they met exclusion criteria: 24 were found to be dialysis patients, 14 patients did not meet criteria for AKI after manual review of their laboratory findings, and 2 were found to not be new admissions (Figure 1). Of the 262 discharge summaries fully reviewed, there was a roughly equal distribution across AKI severity and season (Table 2).

**Table 1.** Total AKI Admissions Identified by Stage and Season.

AKI stage	Discharge season	Number of AKIs	%
Stage 1	January-March	182	15
	April-June	215	17
	July-September	214	17
	October-December	189	15
Stage 1 total		800	65
Stage 2	January-March	67	5
	April-June	77	6
	July-September	68	5
	October-December	52	4
Stage 2 total		264	21
Stage 3	January-March	41	3
	April-June	48	4
	July-September	40	3
	October-December	44	4
Stage 3 total		173	14
Overall		1237	100

*Note*. AKI = acute kidney injury.

**Table 2.** Screened AKI Admissions Divided by Stage and Season.

AKI stage	Discharge season	Number of AKIs	%	Women	Men
Stage 1	January-March	22	8.40	6	16
	April-June	21	8.00	9	12
	July-September	26	9.90	9	17
	October-December	26	9.90	10	16
Stage 1 total		95	36.30	34	61
Stage 2	January-March	17	6.50	11	6
	April-June	24	9.20	11	13
	July-September	23	8.80	7	16
	October-December	26	9.90	10	16
Stage 2 total		90	34.40	39	51
Stage 3	January-March	12	4.60	7	5
	April-June	17	6.50	5	12
	July-September	19	7.30	8	11
	October-December	29	11.10	8	21
Stage 3 total		77	29.40	28	49
Overall		262	100	101	161

*Note*. AKI = acute kidney injury.

### Discharge Summary Review for Presence and Quality of AKI Documentation

The presence of AKI was documented in 53% of the discharge summaries. The AKI documentation was more likely in patients with more severe AKI. Of the patients with AKI stage 3, 75% had AKI documented in their discharge summaries versus 51% in AKI stage 2 (OR, 2.92, *P* = .0015) and 38% in AKI stage 1 (OR, 5.00, *P* < .001; Supplemental Figure S2). Of the 140 total patients with AKI documented, 94 (67%) of discharge summaries referred to the severity of AKI and 116 (83%) documented the status of the AKI at the time of discharge (Supplemental Figure S3).

### Follow-up Plans

**Figure 2.**
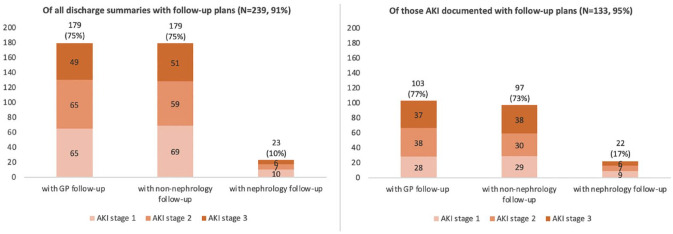
Number of patients with documented follow-up plan in the discharge summary with primary care physician, non-primary care physician, or nephrologist, by stage of AKI. *Note*. The left panel includes all AKI patients’ discharge summaries. The right panel includes discharge summaries that have the presence of an AKI documented. AKI = acute kidney injury; GP = general practitioner.

Some type of follow-up plan was outlined in 239 (91%) of discharge summaries. Of these, 179 (75%) of patients had follow-up with their primary care physician as suggested in the discharge summary. Only 23 (10%) of patients had a plan for a nephrology follow-up recommendation documented. Patients with their AKI documented were more likely to have nephrology follow-up than those without AKI documented (17% vs 10%; Figure 2). In discharge summaries with both AKI and follow-up plans documentation, specific nephrology follow-up plan was documented by AKI severity as follows: 25.0% in AKI stage 1, 15.6% in AKI stage 2, and 11.5% in AKI stage 3 (Figure 2).

### Laboratory Investigations

**Figure 3.**
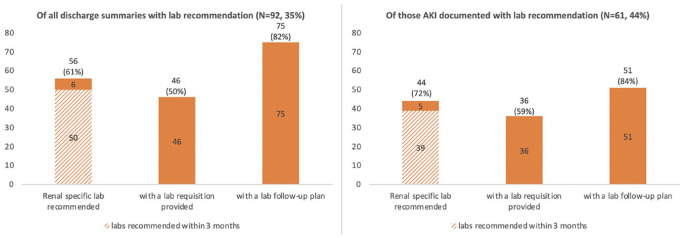
Number of patients with documented laboratory follow-up plans in the discharge summary. *Note*. The left panel includes all AKI patients’ discharge summaries. The right panel includes discharge summaries that have the presence of an AKI documented. AKI = acute kidney injury.

Only 92 (35%) discharge summaries documented recommendations for laboratory investigations after discharge. Patients who had the presence of AKI documented in the discharge summary were more likely to have laboratory recommendations in the discharge summary, compared with patients whose discharge summaries did not document AKI (44% vs 25%, *P* = .0021). Of the 61 discharge summaries with both presence of AKI and laboratory recommendations documented, the majority 44 (72%) made reference to kidney-specific laboratory tests such as for creatinine, estimated glomerular filtration rate (eGFR), electrolytes, or urine studies, and most 51 (84%) recommended that the laboratory tests be followed up within 3 months. In the group of patients with both AKI and laboratory recommendations documented, 36 (59%) made reference to a laboratory requisition being provided to the patient at the time of discharge (Figure 3).

### Medication Management

**Figure 4.**
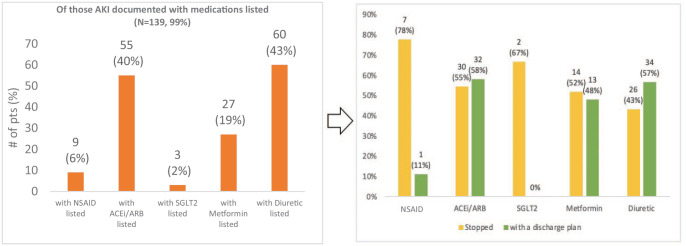
Documentation of medications in the discharge summaries of those with AKI documented (left); percentage of listed medications stopped or with plan in discharge summary (right). *Note*. ACEi/ARB = angiotensin converting enzyme inhibitor/angiotensin II receptor blocker; AKI = acute kidney injury; NSAID = nonsteroidal anti-inflammatory drug; SGLT2 = sodium-glucose cotransporter 2.

Of the discharge summaries that mentioned AKI, 139 (99%) included any form of medication documentation. Of these, 55 (40%) mentioned angiotensin converting enzyme (ACE) inhibitor or angiotensin II receptor blockers (ARBs) and 60 (43%) mentioned diuretics (Figure 4). Other medications commonly held during AKI, such as metformin, nonsteroidal anti-inflammatory drugs (NSAIDs), and sodium-glucose cotransporter 2 (SGLT2) inhibitors were mentioned less commonly (Figure 4). Of those with medications mentioned, the minority mentioned whether that medication had been held or gave specific discharge instructions for that medication (Figure 4).

## Discussion

Our study adds to the limited literature evaluating the quality of discharge communication after AKI. We report incomplete reporting of AKI and unsatisfactory quality of discharge reporting of AKI on medical and cardiac wards in a tertiary care center in British Columbia, Canada. The presence of AKI was mentioned in the discharge summary in just more than half of patients, with documentation being more common in severe AKI. When mentioned, there were substantial deficiencies in completeness of AKI documentation, including severity and trajectory. In addition, follow-up plans regarding laboratory and medication management were often lacking.

Acute kidney injury is strongly associated with adverse outcomes, mortality, reduced quality of life, and increased rehospitalization.^12^ Early intervention and close follow-up after an episode of AKI has been associated with improved outcomes, but these benefits may be lost without effective discharge communication.^5,13^ Unfortunately, previous data suggest that discharge summaries rarely contain complete and reliable documentation regarding the AKI.^8^ This may be one factor that leads to suboptimal follow-up and contributes to poor outcomes in this patient population.

Our findings are similar to those found by Greer et al, in a review of hospital records at Johns Hopkins Hospital in Baltimore, which reported less than half of discharge summaries documenting the presence of AKI.^8^ Strategies are needed for increasing the presence and quality of AKI reporting in discharge summaries. These could include practitioner education regarding the importance of AKI communication, templates for AKI documentation, or even a specific AKI section built into medical discharge summaries to remind physicians to include this information. The latter strategy has been shown to be effective in a UK-based quality improvement initiative.^9^

We found especially low rates of documentation of stage 1 AKI. It is unclear whether these milder forms of AKIs went undetected by the medical team, or were detected but not reported. While severe AKI portends higher risk, even mild AKI episodes are strongly associated with increased risk of both short- and long-term mortality and the development of CKD.^12,13^ Without being aware of these AKI episodes, primary care physicians may be left without critical information needed to optimally care for these at-risk patients. Our findings provide important information that could be used for quality improvement and educational strategies around the importance of detecting and reporting mild AKI.

In those patients with AKI documented, 67% documented the severity, whereas a larger proportion, 83%, mentioned the status or trajectory of the AKI at discharge. Although the latter is encouraging, this still suggests that many discharge summaries make no mention of AKI severity and degree of recovery, which are known to be strong predictors of outcomes following AKI.^14^ Furthermore, the trajectory of the AKI is crucial for practitioners arranging follow-up as it may determine the timing of follow-up and medication management.

It is encouraging that most discharge summaries recommended that patients have follow-up with at least one practitioner, most often with their primary care physician. However, in these cases, it is unclear whether this represents general post-discharge follow-up as opposed to AKI-specific follow-up. Only a minority (17%) of patients had follow-up with a nephrologist recommended. Multiple previous studies have also shown low rates of nephrology referral after AKI, even in patients with severe kidney injury. Siew et al^15^ found that, in patients with severe AKI, only 8.5% had an outpatient nephrology referral following discharge. A retrospective review of US Medicare patients, age 66 years and older, reported that only 13% were seen by a nephrologist within 3 months of discharge.^16^ The benefit of nephrology follow-up after AKI has not been clearly demonstrated. A recent randomized controlled trial of structured nephrology follow-up versus usual care following hospitalization for AKI did not show difference in major adverse kidney outcomes at 1 year, but did result in more timely testing for proteinuria and more consistent in-person visits.^17^ In addition, one cohort study found that nephrology assessment within 3 months of severe AKI was associated with improved mortality.^13^ The potential benefit of nephrology assessment, especially after severe AKI, may stem from expertise in screening for CKD, addressing modifiable risk factors, such as proteinuria, treatment of cardiac risk factors, such as hypertension, access multidisciplinary care resources and patient education, and comfort with medication management in kidney disease. The reason for low nephrology follow-up may be a result of a lack of referral guidelines following AKI, lack of understanding of the benefit, or perception of long specialist wait time. Specific initiatives encouraging nephrology referral, such as dedicated post-AKI clinics with automatic referral, have been shown to increase nephrology follow-up following moderate to severe AKI.^18^ Findings from prior post-AKI interventions have also concluded that the exact mechanism of follow-up may be less important than assuring a post-AKI intervention be flexible, not rely exclusively on in-person clinic visits, and should have the ability to be tailored to individual patient needs.^17,19^

In our review, when AKI was documented, recommendations for outpatient laboratory were made in 44% of discharge summaries. In those with laboratory recommendations, many did not specify whether the laboratory tests were ordered by the discharge team prior to discharge, and whether a requisition was provided. The KDIGO guidelines recommend that creatinine and urine ACR be checked within 3 months of AKI.^7^ A review of Medicare patients in the United States show that only 60% and 10% of patients, respectively, have creatinine and urine ACR checked within 3 months of hospitalization with AKI.^16^ Repeat investigations at 3 months are crucial to screen for the development of CKD and laboratory tests are often required earlier to assess for recovery of AKI and to guide medication management decisions. Clear documentation of this follow-up plan after hospitalization with AKI are key to ensuring that these importance tests occur in the outpatient setting.

Medication management is an important component of AKI and post-AKI management. First, medications that are nephrotoxic or those that can potentiate the damage of volume depletion or hemodynamic changes in kidney perfusion should be held during an episode of AKI. However, many of these medications, such as ACE inhibitors, ARBs, and SGLT2 inhibitors should be restarted when the AKI has recovered to provide their renal, cardiac, and mortality benefits. Although most discharge summaries we reviewed contained a list of discharge medications, many were often lacking crucial information, such as whether the medications were held, the doses changed, and what the discharge plan is for that medication. Cohort studies have shown potential harm when important medications, such as ACEi/ARB are not restarted following AKI. Bidulka et al^20^ found that in patients with an indication for ACEi or ARB and AKI, those with the medication restarted within 30 days of the AKI had a lower risk of 2-year mortality and no increased risk of recurrent AKI or heart failure.

The strengths of this study include a large sample of discharge summaries compared with previous literature. Another strength was the ability to identify all AKIs in the hospital based on laboratory criteria without relying on administrative data, therefore capturing episodes of AKI that may have gone undetected by practitioners. Our study has limitations that require mention. First, our technique for defining admissions and AKI based on laboratory data may have missed or misclassified some patients, such as those directly admitted to the medical ward from places other than emergency, critical care, or the operating room. Second, our definition of AKI based on lab criteria may have missed some of those that met criteria based on urine output. It is also possible that our definition of AKI, based on the highest and lowest creatinine during admission, may have led to some overclassification. In addition, without outpatient laboratory tests, it is possible that we have not captured the true baseline creatinine in some patients. There are also inherent limitations of using data from a single center, which may not be generalizable to other centers with differing care patterns, discharge summary techniques, and medical record systems. Another limitation is that other discharge communications could have occurred outside of a conventional discharge summary (eg, telephone calls). Finally, the study could not logistically access the data to evaluate the extent post-discharge AKI follow-up plans were achieved. This could be explored in future studies about this topic.

## Conclusions

Patients are at high risk of downstream complications after an episode of AKI. Optimizing care for patients following AKI relies on effective communication to outpatient practitioners following hospital discharge. We found suboptimal quality and completeness of discharge reporting in patients hospitalized with AKI. Strategies to improve education and awareness of AKI, as well quality of discharge reporting would be beneficial for this patient population.

## Supplemental Material

sj-docx-1-cjk-10.1177_20543581231222064 – Supplemental material for Assessing Discharge Communication and Follow-up of Acute Kidney Injury in British Columbia: A Retrospective Chart Review Click here for additional data file. Supplemental material, sj-docx-1-cjk-10.1177_20543581231222064 for Assessing Discharge Communication and Follow-up of Acute Kidney Injury in British Columbia: A Retrospective Chart Review by Peter Birks, Bader Al-Zeer, Daniel Holmes, Rami Elzayat, Mark Canney, Ognjenka Djurdjev, Tianyi Selena Shao, Yuyan Zheng, Samuel A. Silver and Adeera Levin in Canadian Journal of Kidney Health and Disease
