# Supplementary material for: Assessing Discharge Communication and Follow-up of Acute Kidney Injury in British Columbia: A Retrospective Chart Review
Source: Can J Kidney Health Dis. 2024 Feb 5;11:20543581231222064. doi: 10.1177/20543581231222064 (PMC10845986; doi:10.1177/20543581231222064)
Supplement: sj-docx-1-cjk-10.1177_20543581231222064 – Supplemental material for Assessing Discharge Communication and Follow-up of Acute Kidney Injury in British Columbia: A Retrospective Chart Review [file sj-docx-1-cjk-10.1177_20543581231222064.docx]

**Supplement:**

Supplement figure 1. AKI discharge summary data collection tool.

Supplement figure 2:

Proportion of patients with AKI documented in discharge summary by stage of AKI.


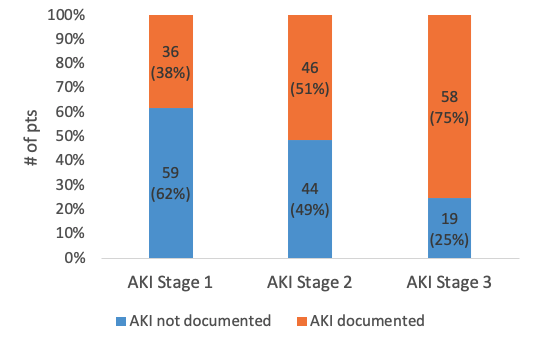


Supplement figure 3.

Proportion of patients with AKI severity and AKI status at discharge documented in discharge summary (of those patients with the AKI documented in the discharge summary).
